# Supplementary material for: Function of the Porcine TRPC1 Gene in Myogenesis and Muscle Growth
Source: Cells. 2021 Jan 13;10(1):147. doi: 10.3390/cells10010147 (PMC7828378; doi:10.3390/cells10010147)
Supplement: Supplementary file 1 [file cells-10-00147-s001.pdf]

# Supplementary Information

**Supplementary Table S1. The primer sequences for SqRT-PCR and qRT-PCR**

| Species | Genes | Primer sequences (5′ to 3′) |
|---|---|---|
| Pig | *TRPC1* | F: CATCCAAAGGCAAGGTTA |
| | | R: AAGTCCGAAAGCCAAGTA |
| | *β-actin* | F: GCCAACCGTGAGAAGATGACT |
| | | R: GTGACCCCATCCCCAGAGT |
| Mouse | *Ki67* | F: ATCATTGACCGCTCCTTTAGGT |
| | | R: GCTCGCCTTGATGGTTCCT |
| | *Cyclin B* | F: AATACCTACAGGGTCGTGAAGTGA |
| | | R: GCTGTATCATCTTCTTGGGCAC |
| | *CDK4* | F: GCTGCTACTGGAAATGCTGACC |
| | | R: AGCCTTGGGGGGAAACAGA |
| | *p27* | F: CAGGCAAACTCTGAGGACCG |
| | | R: TCGGGGAACCGTCTGAAAC |
| | *BAD* | F: GCTTAGCCCTTTTCGAGGAC |
| | | R: GATCCCACCAGGACTGGAT |
| | *MyoD* | F: CGAGCACTACAGTTGGCGACTAAGAT |
| | | R: GCTCCACTATGCTGGACAGGCAGT |
| | *MyoG* | F: CCATCCAGTACATTGAGCGCCTACA |
| | | R: ACGATGGACGTAAGGGAGTGCAGAT |
| | *MyHC* | F: CAAGTCATCGGTGTTTGTGG |
| | | R: TGTCGTACTTGGGCGGGTTC |
| | *Myomaker* | F: ATCGCTACCAAGAGGCGTT |
| | | R: CACAGCACAGACAAACCAGG |
| | *β-1integrin* | F: TTACAAGAGTGCCGTGACAACTG |
| | | R: GACTAAGATGCTGCTGCTGTGAG |
| | *Atrogin1* | F: GCAGCTGGATTGGAAGAAGA |
| | | R: GAGCAGCTCTCTGGGTTGTT |
| | *Bmp4* | F: CCGGATTACATGAGGGATCT |
| | | R: CCTGGGATGTTCTCCAGATG |
| | *Murf* | F: GCAAGGCTTTGAGAACATGG |
| | | R: TCTTCCTCATCAGCCTCCTC |
| | *Foxo3* | F: ACAAACGGCTCACTTTGTCC |
| | | R: CTGTGCAGGGACAGGTTGT |
| | *Fst* | F: TCTCTGCGATGAGCTGTGTC |
| | | R: CCTCCTCTTCCTCCGTTTCT |

| | |
|---|---|
| *Nog* | F: TGTGGTCACAGACCTTCTGC |
| | R: GTGAGGTGCACAGACTTGGA |
| *LRP6* | F: CGTATGAAGGGAGACGGGGA |
| | R: CTTTCTGTGGCAGGCGATGG |
| *LEF1* | F: ATGATTCCTGGTCCCCCTG |
| | R: GCTCCTGCTCCTTTCTCTGTTC |
| *TCF1* | F: TGGACATTGACATTCGCATT |
| | R: CACACGGTCAGTCCATGTTC |
| *CCND* | F: GCCCTCCGTATCTTACTTCAAG |
| | R: ACCTCCTCTTCGCACTTCTG |
| *Axin2* | F: GCTGACGGATGATTCCATGT |
| | R: ACTGCCCACACGATAAGGAG |
| *GSK3β* | F: TTTTTCGATGAATTGCGGG |
| | R: TTATTGGTCTGTCCACGGTCTC |
| *SPFR2* | F: ACCCTTTGTAAAAATGACTTCGCA |
| | R: GATTTCTTCAGGTCCCTTTCGG |
| *SPFR4* | F: CAAGTCTTTGTCACCTATCCCTCG |
| | R: GTGCGGCTGGCTATCTGCTT |
| *TRPC1* | F: GTGGTATGAAGGGTTGGAAGACT |
| | R: TCTGCTACAAGCGTGGGGTG |
| *β-actin* | F: ATCTGGCACCACACCTTCTACA |
| | R: AAGGTCTCAAACATGATCTGGGT |

**Supplementary Table S2. Sequence, targets*, and amplicon size of the primers used for SNP identification of the pig TRPC1*** **gene**

| Primers | Target regions (bp) | Primer sequences (5′ to 3′) | Amplicon sizes (bp) |
|---|---|---|---|
| *TRPC1*-P1 | -2142/-1548 | F: CTAGAGTTGTGATGGGTCTTC | 595 |
| | | R: GCTCATTGTAAATCTGTGGC | |
| *TRPC1*-P2 | -1702/-942 | F: TTACCCCTAAACACTCTGG | 761 |
| | | R: CATTGGGATCGGCTCTA | |
| *TRPC1*-P3 | -1121/-662 | F: CAAGCACTAAGGGGACA | 550 |
| | | R: GGTTGCAGGTTTGAGGTA | |
| *TRPC1*-P4 | -678/+121 | F: ACCTCAAACCTGCAACC | 800 |
| | | R: ACCTCCCGCACATCCTT | |

**Supplementary Table S3. The sequences of *TRPC1* siRNA fragments**

| siRNA | Oligo sequences (5′ to 3′) |
|---|---|
| si-NC | GGGUCCAUUACAGAUUUCATT |
| | UGAAAUCUGUAAUGGACCCTT |
| si-682 | CGACAAGGGUGACUAUUAUTT |
| | AUAAUAGUCACCCUUGUCGTT |
| si-886 | GGGAGCUGUUGAUAUACUATT |
| | UAGUAUAUCAACAGCUCCCTT |
| si-1220 | CUGAGAGCGUUUGAACUUATT |
| | UAAGUUCAAACGCUCUCAGTT |

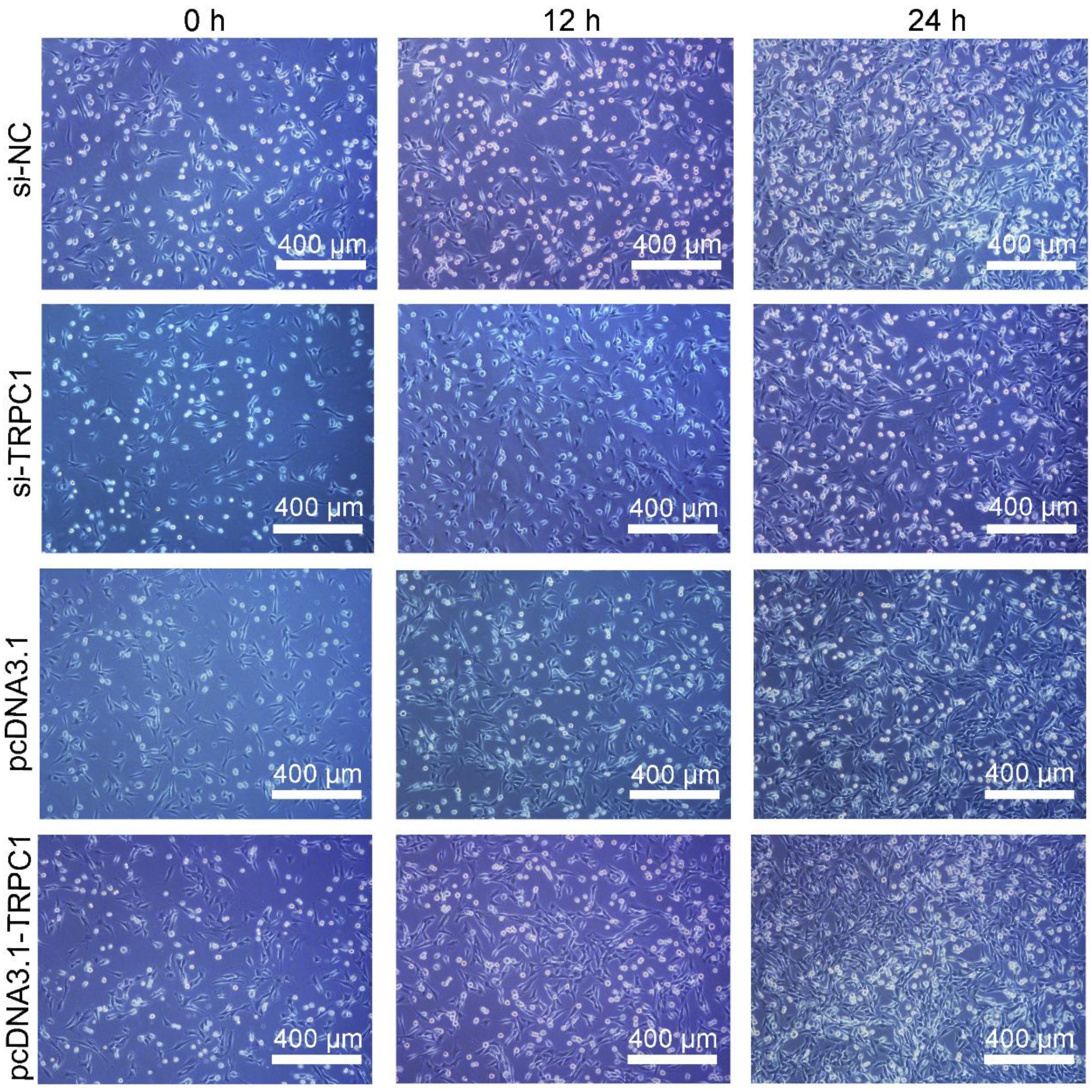


**Supplementary Fig. S1 The microscopic images of proliferated cells.** C2C12 with different treatments at 0 h, 12 h and 24 h, respectively, *n* = 3, scale bar = 400 μm.

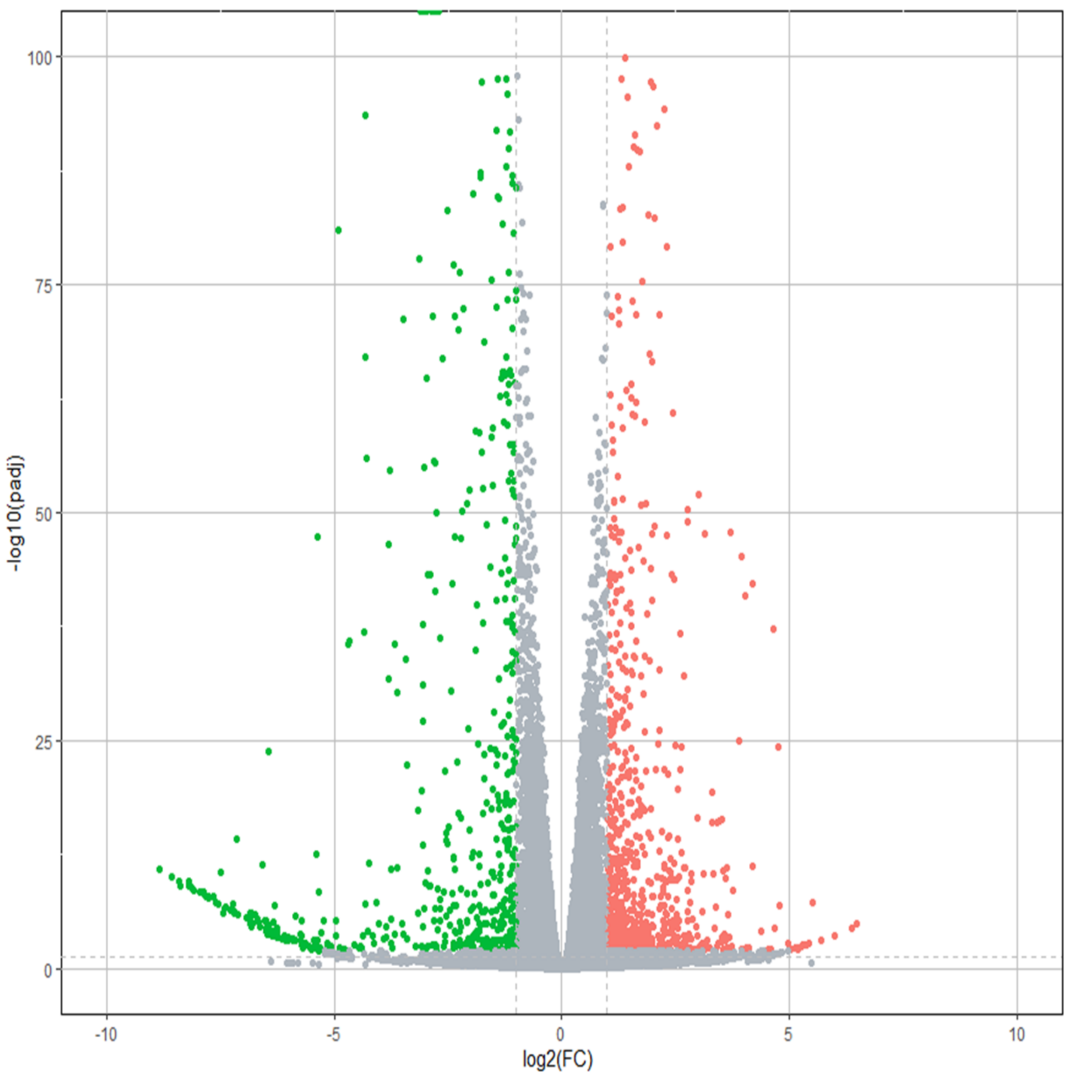


**Supplementary Fig. S2 Differentially expressed genes identification. Volcano plot displaying DEGs between *TRPC1*-overexpression group and control.** Upregulated and downregulated genes are shown in red and green, respectively. Gray dots represent genes with similar expression levels.

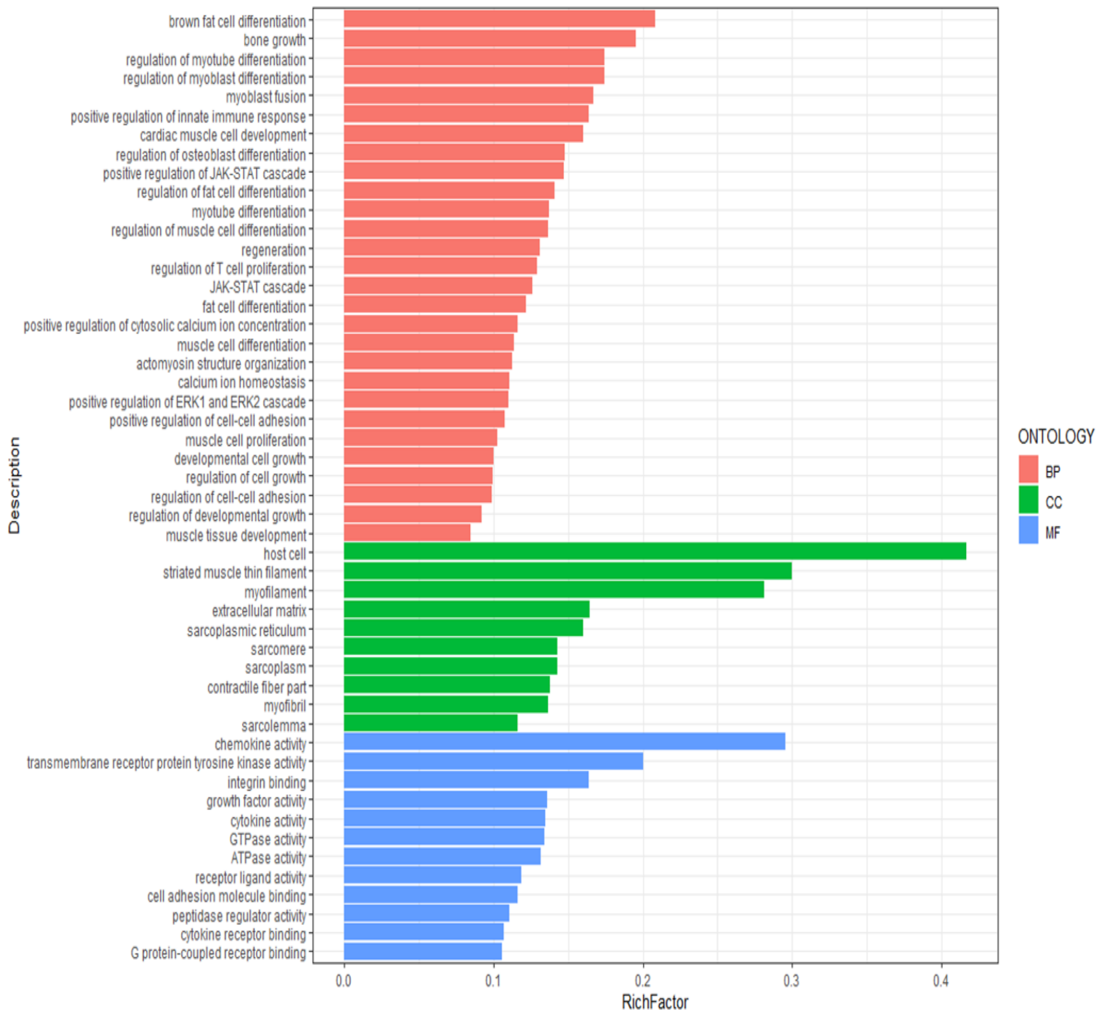


**Supplementary Fig. S3 Significantly enriched GO terms of differentially expressed genes.** The green clusters represent the cellular component, the red clusters represent the biological process, and the blue clusters represent the molecular function of the GO terms.

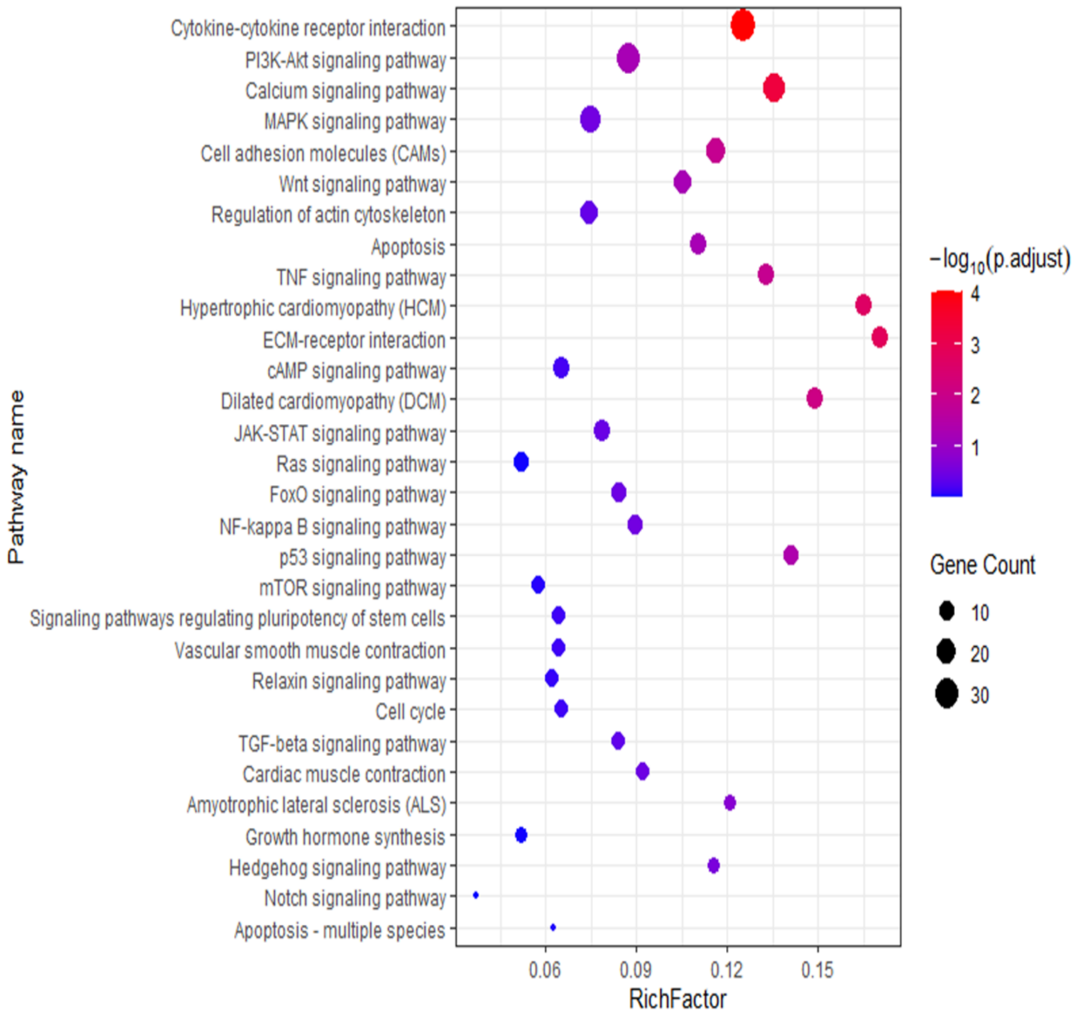


**Supplementary Fig. S4 KEGG-enriched scatter plot of DEGs between control and *TRPC1*-overexpressed C2C12.** The rich factor is the ratio of DEG numbers annotated in this pathway term to the total gene numbers annotated in this pathway term. The smaller the P-value, higher the significance.
